# Medical History from the Earliest Times

**Published:** 1892-09-10

**Authors:** E. T. Withington


### Medical History from the Earliest Times.

By E. T. Withington, M.A., M.B. Oxon.

### XXIII.—ROMAN MILITARY MEDICINE.

When the fifth cohort of the "Vigiles" set up a pillar in honour of the Emperor Caracalla (A.D. 212) they not only immortalised themselves, but gave us some important information on Roman military medicine, for besides the names of 13 officers and about 1,000 privates, we find those of four physicians, C. R. Hilaris, C. J. Hermes, L. F. Pollux, and S. L. Ecarpus. Another pillar set up at the same time by their comrades of the second cohort has been nearly destroyed, but the list of officers still remains, and concludes with the four "medici," Cl. Thamyra, Fl. Panmene, Aur. Hegumene, and Aphrodite (?) It is interesting to note that six out of the eight names are Greek, and that in the second inscription they are put in the vocative case, as if the stone mason, being ignorant of the language, had copied them direct from the roll-call of the cohort.

But before describing the well-developed medical service revealed by these and other records, let us take a glance at the state of Roman military surgery in earlier times. The armies of the Republic were levies of citizens, and we have seen that the Roman citizen of that age considered medicine beneath his dignity. It is therefore not surprising to find no mention of army surgeons, but it by no means follows that the wounded were entirely uncared for. The soldiers themselves possessed some rude surgical skill, and as in modern armies, each seems to have carried bandages, and perhaps other appliances, with him, for we are told that on one occasion a large number of soldiers bandaged various parts of their bodies and pretended they were wounded in order to avoid serving under an unpopular leader. A victorious army is unable to pursue the enemy, "because it halted to collect its wounded," and a defeated one carries off with it as many wounded as possible; what became of the rest we had better not inquire. Livy further informs us that the wounded when brought home were cared for in the houses of the great, and that the Fabian family made themselves especially popular by their zeal in this good work. On one occasion the chief officers received them in their own tents after a battle, and had them tended there, doubtless by slaves, some of whom may have been of Greek origin, and have had special medical training. Towards the close of the Republic we find distinguished generals, such as Cæsar, the younger Cato, and the Consul Pansa always attended by Greek physicians, and there are even indications of an Ambulance service, for Labienus, after the battle of Ruspina, sent his wounded "in waggons" to Adrumentum.

Cæsar's grant of the citizenship to all physicians practising in Rome may have been preliminary to the establishment of an Army Medical Service, for none but citizens could serve in the legions, and (as is shown by the well-known story of how Cæsar quelled a mutiny) the haughty soldier considered even that coveted title a degradation. At any rate the existence of a standing army must soon have made the introduction of some definite medical system a matter at once necessary and politic. The safety of a despot depends much on his influence over the army, and we find Tiberius, the most crafty of the Emperors, winning the favour of the troops during his campaign in Illyria by taking with him physicians, litters, and even a bath for the special benefit of the wounded. The development of an Army Medical Service would thus be favoured by both bad and good rulers, and the following is a brief outline of its condition under the most warlike of the latter class, the Emperor Trajan, at the close of the first century.

The Roman army of this period may be classed under three heads: (1) The garrison of the city, comprising nine Pretorian and four urban cohorts, together with the seven cohorts of the Vigiles, who acted as police and firemen. Each cohort contained 1,000 to 1,500 men, and each appears to have been provided with four surgeons or "medici." The Pretorians seem to have even had specialists, for we hear of a "medicus clinicus," or pure physician, attached to one of their cohorts. (2) Thirty legions, each consisting of ten cohorts and about 6,500 men. Here we only find mention of a "medicus legionis," but from what is known of the very liberal provision for the more stay-at-home soldiers, it would be absurd to suppose that there was only one surgeon for each legion, and we may estimate their number as at least six, or perhaps ten, though they were not attached to particular cohorts. They ranked with the standard-bearer and trumpeters among the "principales" or non-commissioned officers, and, as may be seen on Trajan's column, they wore the ordinary dress and arms of the legionary. (3) The infantry and cavalry of the allies, which were divided into cohorts and alae respectively. Their surgeons were, perhaps, of a somewhat inferior class and are sometimes distinguished by the title "ordinarius." Thus, the Newcastle Museum contains the tombstone of Anicius Ingenuus, "medicus ordinarius" to the first Tungrian cohort, one of those employed in building and defending the Roman wall.

With the standing army came the stationary camps, with which we may probably connect the camp surgeon "medicus castrensis," and the "valetudinarium," or military hospital. The latter is first mentioned by Hyginus in the second century, but had probably already existed for some time. He recommends that it should be placed opposite the "Veterinarium," but at a sufficient distance to save the patients from being disturbed by the noise of the "fabrica," or blacksmith's shop. When more than three legions were in the camp, there were additional valetudinaria, and their general arrangements were superintended by special officers, the "Optiones Valetudinarii," who ranked after the Centurions.

The system thus briefly sketched was continued during the Byzantine Empire, with some slight modifications, and with the interesting additions of the "deputati" and the "nosocomi." The "deputati" are first mentioned in the Emperor Maurice's book "On Tactics" (about A.D. 590), but, as he modestly confesses that he knows nothing of the subject, and has copied from older writers, they may have originated somewhat earlier. In every troop or "bandon" of two to four hundred men, eight or ten stout fellows were deputed to ride immediately behind the fighting line to pick up and rescue the wounded, for which purpose their saddles had two stirrups on the left side, while they themselves were provided with water-flasks, and perhaps applied temporary bandages. They were encouraged by a reward of a piece of gold for each man they rescued. The "nosocomi" were male nurses attached to the military hospitals, but not inscribed "on the strength" of the legions, and were probably for the most part of the servile class.

The Roman navy, though considered a decidedly inferior branch of the service, was equally well provided with surgeons, and we possess the epitaphs of at least four naval "medici" of the triremes "Cupid," "Tiger." "Faith," and of one unnamed vessel respectively. Three out of the four have the epithet "duplicarius," indicating that they received double pay, and the fourth died a few weeks after his appointment to the "Faith." The privilege can scarcely have been due to the amount of their work, for the small "Liburnians" which composed the Roman fleet did not contain more than about two hundred men, including rowers and soldiers; but we may perhaps attribute it to the dislike which the ancients, and especially the Romans, had of the sea, and which may have rendered it necessary to offer special inducements to candidates.

